# Characterization of gut microbiota dynamics in an Alzheimer’s disease mouse model through clade-specific marker-based analysis of shotgun metagenomic data

**DOI:** 10.1186/s13062-024-00541-7

**Published:** 2024-10-30

**Authors:** Francesco Favero, Angela Re, Mohammed Salim Dason, Teresa Gravina, Mara Gagliardi, Marta Mellai, Marco Corazzari, Davide Corà

**Affiliations:** 1 Department of Translational Medicine (DIMET), University of Piemonte Orientale, Via Solaroli 17, I-28100 Novara, Italy; 2 Center for Translational Research on Autoimmune and Allergic Disease (CAAD), C.so Trieste, 15/A, I-28100 Novara, Italy; 3 Department of Applied Science and Technology (DISAT) - Politecnico di Torino, C.so Duca degli Abruzzi, 24, I-10129 Torino, Italy; 4 Department of Health Sciences (DISS), University of Piemonte Orientale, Via Solaroli 17, I- 28100 Novara, Italy

**Keywords:** Shotgun metagenomics, Alzheimer’s disease, 3xTgAD mice, Gut microbiota, MetaPhlAn 4

## Abstract

Alzheimer’s disease (AD) is a complex neurodegenerative disorder significantly impairing cognitive faculties, memory, and physical abilities. To characterize the modulation of the gut microbiota in an in vivo AD model, we performed shotgun metagenomics sequencing on 3xTgAD mice at key time points (i.e., 2, 6, and 12 months) of AD progression. Fecal samples from both 3xTgAD and wild-type mice were collected, DNA extracted, and sequenced. Quantitative taxon abundance assessment using MetaPhlAn 4 ensured precise microbial community representation. The analysis focused on species-level genome bins (SGBs) including both known and unknown SGBs (kSGBs and uSGBs, respectively) and also comprised higher taxonomic categories such as family-level genome bins (FGBs), class-level genome bins (CGBs), and order-level genome bins (OGBs). Our bioinformatic results pinpointed the presence of extensive gut microbial diversity in AD mice and showed that the largest proportion of AD- and aging-associated microbiome changes in 3xTgAD mice concern SGBs that belong to the Bacteroidota and Firmicutes phyla, along with a large set of uncharacterized SGBs. Our findings emphasize the need for further advanced bioinformatic studies for accurate classification and functional analysis of these elusive microbial species in relation to their potential bridging role in the gut-brain axis and AD pathogenesis.

**Supplementary Information:**

The online version contains supplementary material available at 10.1186/s13062-024-00541-7.

## Background

Alzheimer’s disease (AD) is a prevalent neurodegenerative disorder clinically characterized by the progressive degeneration of cognition, memory, and motor abilities [1]. Despite extensive research, its etiology remains largely elusive. Classical pathological hallmarks include accumulations of the amyloid-β (Aβ) peptide [2] and intracellular neurofibrillary tangles composed of hyperphosphorylated tau protein [3]. These protein aggregations are associated with disruptions in autophagic flux due to cathepsin B S-nitrosylation and alterations in the ubiquitin-proteasome system [4], which may further exacerbate cellular dysfunction. Building on these findings, recent studies conducted on AD mouse models have shown an upregulation of pro-apoptotic factors, leading to increased cell death compared to control mice [5]. Concurrently, other research has reported that mitochondrial dysfunction, resulting from knockout (KO) of CEND1—a gene critical for neurogenesis—contributes to cognitive impairments [6]. Furthermore, Caspase-6 and pathways involving NIrp1 and Caspase-1 have been implicated in AD pathogenesis by promoting neuroinflammation [7, 8], suggesting a complex interplay of molecular alterations that drives AD progression.

More recently, the scope of AD research has expanded to include changes in the gut microbiota as potential disease biomarkers and/or disease modifiers [9]. Humans host complex microbial communities, mainly residing in the gut, which produce a range of biomolecules crucial for systemic functions [10], such as the biosynthesis of vitamins, protection from pathogen overgrowth, digestion of dietary components, and immunomodulation [11, 12]. This is supported by emerging evidence pointing to the existence of a microbiota-gut-brain axis [13–16], suggesting a link between changes in the gut microbiome and various neurological conditions [17–19]. However, despite extensive microbiome profiling studies in AD patients [20, 21] and AD mouse models [22–25], no specific microbial signature has been consistently identified for this disease. Nonetheless, some evidence does suggest shifts in microbiota composition in individuals with AD [26] and AD mouse models [4]. Furthermore, emerging research indicates that modulating the gut microbiota composition—through germ-free conditions, antibiotics, or significant dietary changes—can influence the progression of AD pathology [27–30] and cognitive decline [31–33] in genetic models of this disease.

Against this backdrop, the primary goal of studies investigating the relationship between the microbiota and AD is that of ideally formulating hypotheses connecting the functions of specific bacterial species to human health, to then determine how these functions differ between AD patients and healthy individuals [34]. A critical requirement for these studies is the availability of high-fidelity and high-throughput genomic characterization of microbial biomass in experimentally relevant model systems. However, despite the usefulness of transgenic mice in preclinical AD research [35–38], no comprehensive survey of the entire gut microbiota in AD models using shotgun metagenomics has yet been conducted. Indeed, most studies regarding the structural characteristics of gut microbiome in AD mouse models, including our previous 16S genomic analysis of stool samples from 3xTgAD mice [39], have relied on the application of 16S rRNA gene amplicon sequencing [20, 21, 23–25, 40]. Despite its widespread use, this method has limitations, particularly in its ability to capture the full diversity of microbial species present in a sample. Conversely, high-resolution shotgun metagenomics—seldom applied for AD gut microbiome profiling [26]—allows for a more comprehensive genomic analysis. This approach is often underutilized mostly due to the lack of complete reference genomes that can account for all members of the mouse microbiome [41, 42].

To address this gap and gain deeper insights into the role of the gut microbiota in AD, here we have performed high-resolution and high-throughput genomic profiling of microbial biomass in 3xTgAD mice—an in vivo model that recapitulates the progression of AD—compared to age-matched WT littermates, using shotgun metagenomics followed by a clade-specific marker-based bioinformatic analysis [43, 44]. Our results highlight the challenges posed by unknown taxa, revealing significant functional diversity within AD-associated microbial communities.

## Methods

### Mouse model

Fecal samples were collected from homozygous 3xTgAD mice (*n* = 6, for each timepoint) carrying all three mutant alleles [B6;129-Psen1tm1MpmTg (APPSwe, tauP301L)1Lfa/Mmjax1] and wild-type (WT) mice (*n* = 6, for each timepoint) (B6129SF2/J), which served as controls.

### Sample Collection, Library Preparation and Shotgun sequencing

Fecal pellets were collected from each mouse at two months (T1 = 2 mos), six months (T2 = 6 mos) and twelve months (T3 = 12 mos). Samples were placed on dry ice until sampling was completed and subsequently stored at − 80 °C until further processing.

The stool samples were then thawed at room temperature, and microbial DNA was isolated using the QIAmp^®^ PowerFecal^®^ Pro DNA isolation kit (Qiagen NV, Hilden, Germany), according to the manufacturer’s instructions. The yield and quality of bacterial DNA were determined on a NanoDrop™ 2000 spectrophotometer (Thermo Fisher Scientifics Inc., Waltham, MA, USA). The quantity was assessed using an InvitrogenTM Qubit™ 1X dsDNA HS Assay Kit (Invitrogen Co., Thermo Fisher Scientific Inc.) on a Qubit 4 fluorometer (Invitrogen Co.).

Whole genome shotgun libraries were built using the DNA Library Prep kit (Illumina Inc., San Diego, CA, USA), as per the provider’s instructions. Briefly, the extracted DNA (250 ng) was fragmented using Bead-Linked Transposomes (BLTs) and amplified through limited-cycle PCR with Nextera DNA Combinatorial Dual (CD) Indexes for multiplexing. After amplification, the libraries were cleaned, quantified fluorometrically, and analyzed for quality on a 4200 TapeStation system via a High Sensitivity D5000 ScreenTape assay (both from Agilent, Santa Clara, CA, USA). Each library was normalized to an equimolar concentration before pooling, and the resulting mixture was sequentially diluted and denatured along with phase correction (PhiX Control v3, Illumina).

Shotgun metagenomics data were generated from the collected fecal samples at the Facility of Genomics&Transcriptomics at the Research Center on Autoimmune and Allergic Diseases (UPO-CAAD) in Novara, Italy. Sequencing was carried out on an Illumina NextSeq^®^ 550 platform using a NextSeq^®^ High Output v2.5 Reagent Kit (Illumina) for a 2 × 150 paired-end sequencing.

### Sequence Quality Control

Quality control (QC) reports for the shotgun dataset were generated using the MultiQC software [45] version 1.14. The high quality of the sequencing reads and absence of adapter sequences confirmed that no further quality processing was necessary.

### Host DNA removal and taxonomic profiling

The mouse (host) DNA reads in the dataset were removed using Bowtie 2 with default parameters [46] by mapping paired-end reads against the *Mus musculus* reference genome (GRCm38/mm10) and discarding the aligned reads. Furthermore, reads with a nucleotide percentage > 80% of nucleotides as “G” or “N” were filtered out along with their paired ends. The non-host reads that failed to map to the host reference genome were retained for downstream processing.

For taxonomic profiling of microbiome samples, MetaPhlAn 4 (version 4.1.0) [43] was employed on non-host reads using the database version mpa_vJun23_CHOCOPhlAnSGB_202307 to analyze every taxon up to the species level. MetaPhlAn 4 uses an integrated database of microbial genomes and metagenome-assembled genomes (MAGs), which allows the algorithm to derive a set of species-level genome bins (SGBs), enabling accurate detection and quantification of microbial species in metagenomic data [47]. An SGB is defined by clustering the microbial species spanning at most 5% genetic diversity [47]. A taxonomic label is assigned to each SGB based on the presence of characterized genomes from isolate sequencing. Thus, SGBs obtained from MetaPhlAn 4 are divided into known SGBs (kSGBs) and unknown SGBs (uSGBs). kSGBs receive a taxonomic label based on the species of the reference genomes within the bin, whereas uSGBs, lacking reference genomes, are assigned up to the closest related phylum, with genus-level and family-level annotations provided when possible.

### Data normalization and statistical analysis

Differential abundance of taxa across AD time points and between AD and WT samples was determined from the raw read counts associated with each taxon as determined by the MetaPhlAn 4 algorithm. Initially, only taxa with a prevalence ≥ 10% were retained for subsequent analysis. Taxa were normalized using “geoMeans” from the DESeq2 normalization method [48]. The DESeq2 algorithm was then used to assess differential taxa using test="Wald” and fitType="parametric” options, with |log2FC| > 1 and Benjamini–Hochberg FDR-adjusted p-value < 0.05 as statistical thresholds.

For alpha diversity analysis, the Vegan R package 2.6-4 [49] was used to calculate both Simpson’s and Shannon’s indexes for taxa with a prevalence of ≥ 10%. Moreover, the Total Scum Scaling (TSS) normalization method was performed using the “decostand” function, scaling the data by a factor of 1e7. The Shannon’s and Simpson’s indexes were calculated using the “diversity” function in the Vegan package. The Wilcoxon Rank-Sum test was used to identify statistically significant differences (wilcox.test, p-value < 0.05).

For beta diversity analysis, the Vegan package was employed in a similar manner to alpha diversity calculations, excluding the TSS normalization and data scaling adjustments. Beta diversity was assessed using the Bray-Curtis distance calculated by the “vegdist” function with method set to “bray”. Differences between groups were statically analyzed using the “adonis2” function to perform the Adonis test (p-value < 0.05).

Relative abundance values reported in figures were determined using the MetaPhlAn 4 algorithm. The annotation of selected marker genes was performed using the online version of EggNOG-mapper v2 with default parameters [50].

## Results

**Fig. 1:**
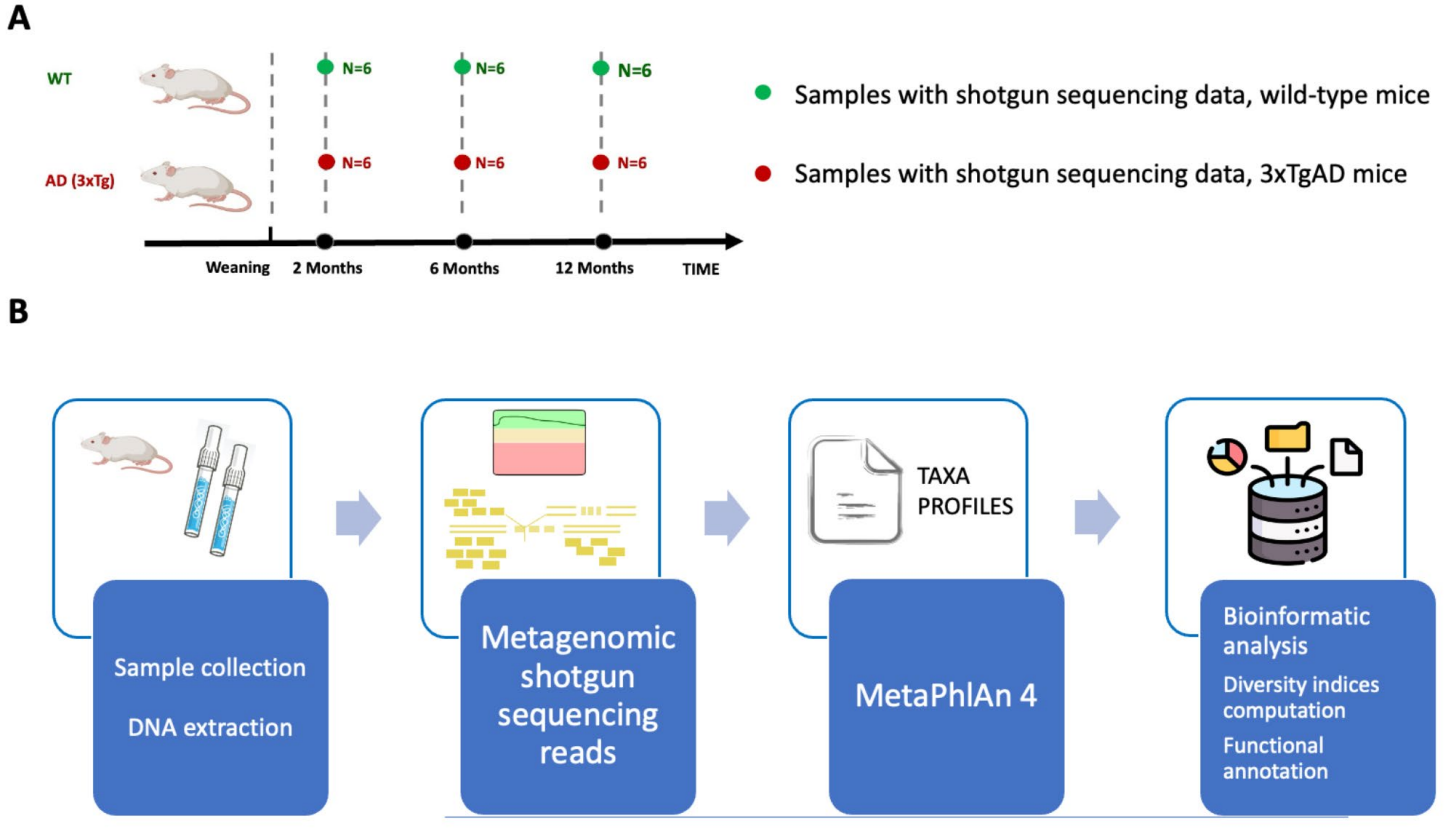
Study design and experimental analysis framework. (**A**) Study design showing the experimental groups and time points for sample collection. WT and AD (3xTg) mice were sampled at two months (T1), six months (T2), and twelve months (T3). At each time point, a total of six samples (*n* = 6) were collected for both WT and AD mice. (**B**) Workflow illustrating the bioinformatics pipeline employed in the analysis

To investigate how the intestinal microbiome is regulated during AD development and how it differs between the AD and healthy states, we systematically examined age-related changes detected by shotgun metagenomics in the microbiota composition of 3xTgAD mice and their age-matched WT littermates at two months (T1 = 2 mos), six months (T2 = 6 mos), and twelve months (T3 = 12 mos). We compared the microbiota profiles of AD vs. WT mice at each time point (AD vs. WT at T1; AD vs. WT at T2; AD vs. WT at T3) and investigated the longitudinal changes in the AD samples over time (AD_T1 vs. AD_T2; AD_T1 vs. AD_T3; AD_T2 vs. AD_T3). A schematic of the study design is reported in Fig. 1A, while the analysis workflow is outlined in Fig. 1B. A list of the total number of reads obtained for each sample, along with the relative non-host reads, is provided in Table S1.

### Profiling of mouse gut microbiome shows the prevailing presence of uncharacterized microbial species

**Fig. 2:**
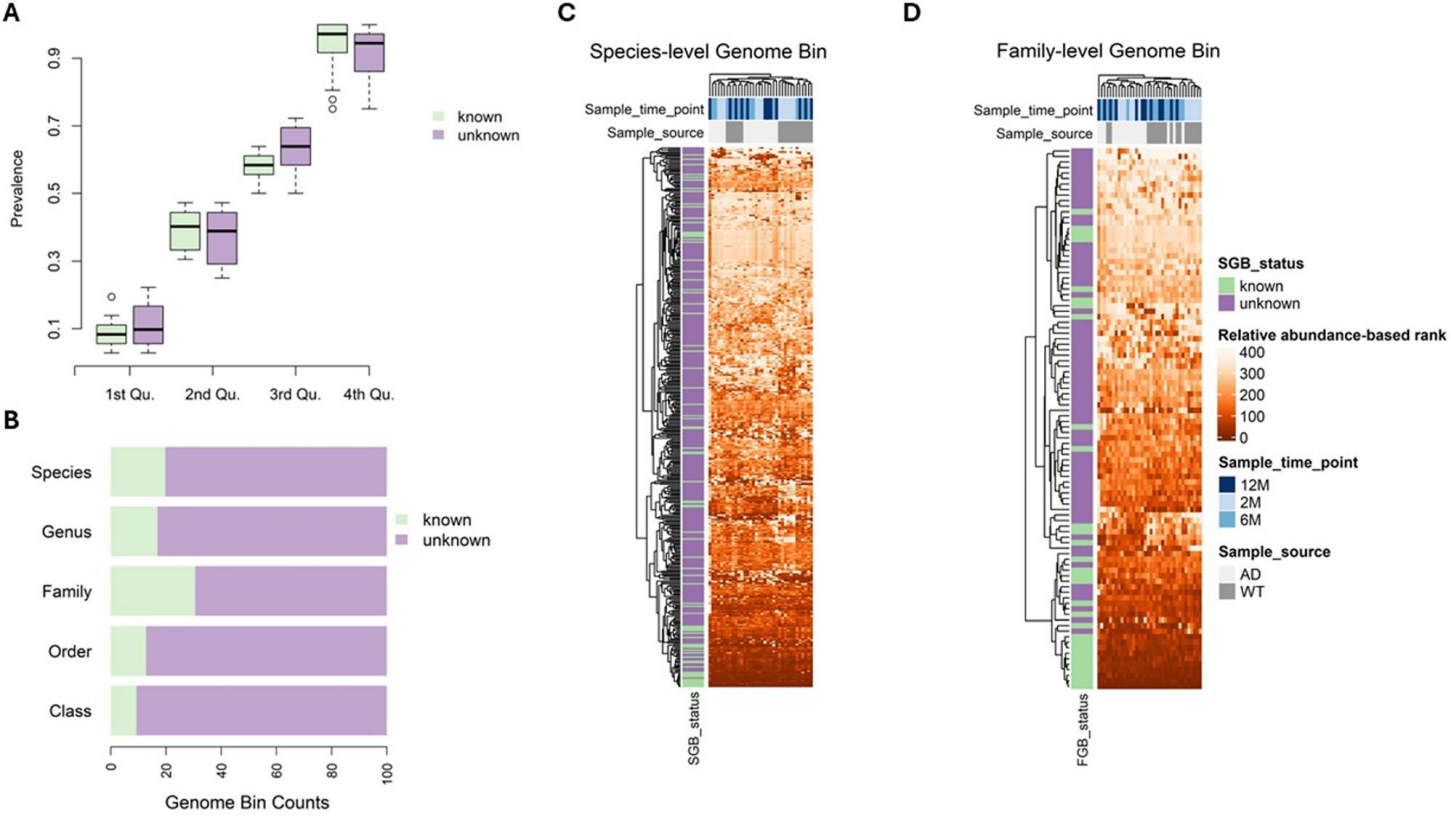
Prevalence of unknown taxa in MetaPhlAn 4 profiling of the mouse gut microbiome. (**A**) Taxonomically unlabeled species-level genome bins (SGBs) are widely distributed though the dataset samples. SGBs are categorized into quartiles according to their prevalence across the samples included in the dataset, and the proportions of known and unknown SGBs (kSGBs and uSGBs, respectively) in each quartile are comparatively assessed. kSGBs prevail over uSGBs (test of equal proportions, statistical significance set at p-values < 0.05) exclusively in the fourth quartile, which includes SGBs with prevalence ranging from 75 to 100% of samples. Conversely, in the third quartile, uSGBs dominate over kSGBs. (**B**) At taxonomic levels higher than species, the fraction of the mouse gut microbiome that remains taxonomically uncharacterized is substantial. The panel depicts the fractions of known and unknown genome bins detected across the entire dataset for each taxonomic level: species, genus, family, order, and class. (**C**, **D**) Taxonomically annotated SGBs tend to display higher relative abundance compared to SGBs that currently lack taxonomic annotation. The relative abundance of each genome bin is averaged across either AD or WT samples collected at each time point, and these average relative abundances are then converted into ranks for each time point, with higher average values corresponding to higher ranks. The heatmaps show the ranks for SGBs (C) and family-level genome bins (FGBs, D) at each sampled time point. Color codes shown in legend help distinguish known from unknown genome bins, AD from WT samples, as well as sampling time points

MetaPhlAn 4 identified and quantified 385 SGBs, comprising 76 kSGBs and 309 uSGBs. We then evaluated single SGB prevalence across samples, finding no statistically significant difference in the proportion of kSGBs vs. uSGBs with > 50% prevalence. Of note, the proportion of uSGBs was significantly higher than that of kSGBs in the 50–75% prevalence range (test of equal proportions, p-value = 0.003), whereas kSGBs predominated in the 75–100% prevalence range test of equal proportions, p-value = 0.02) (Fig. 2A). Thus, these differential prevalence rates underscore the importance of accessing and characterizing the unknown fraction of the SGBs in the AD gut microbiome.

The mapping challenges of microbial samples in our dataset against isolate genomes extended beyond the species level. About 70% of the family-level genome bins (FGBs)—similar to SGBs but with up to 30% nucleotide divergence—remained uncharacterized in more than half of the samples. Further analysis of the taxonomic profiles at higher ranks revealed that the genome bins yet to characterized were more prevalent than the characterized ones across the genus, family, order, and class levels (Fig. 2B).

Next, we evaluated the average relative abundance of each SGB at each observation time point and subsequently ranked them based on their abundance. As expected, kSGBs featured higher relative abundances than uSGBs (SGB, Kolmogorov-Smirnov test, p-value = 0.0004), with relative abundances calculated as the average value for the samples collected at each time point (Fig. 2C). This pattern persisted across higher taxonomic levels (FGB, Kolmogorov-Smirnov test, p-value = 0.0005) (Fig. 2D).

Table S2 provides a comprehensive overview of the abundance of each taxon measured by MetaPhlAn 4.

### Composition of the gut microbiome in AD vs. WT mice

The dataset of SGBs quantified in the AD or WT microbiomes sampled at the set time points consisted of 385 SGBs classified into 301 genera, 98 families, 78 orders, 75 classes, and 9 phyla.

**Fig. 3:**
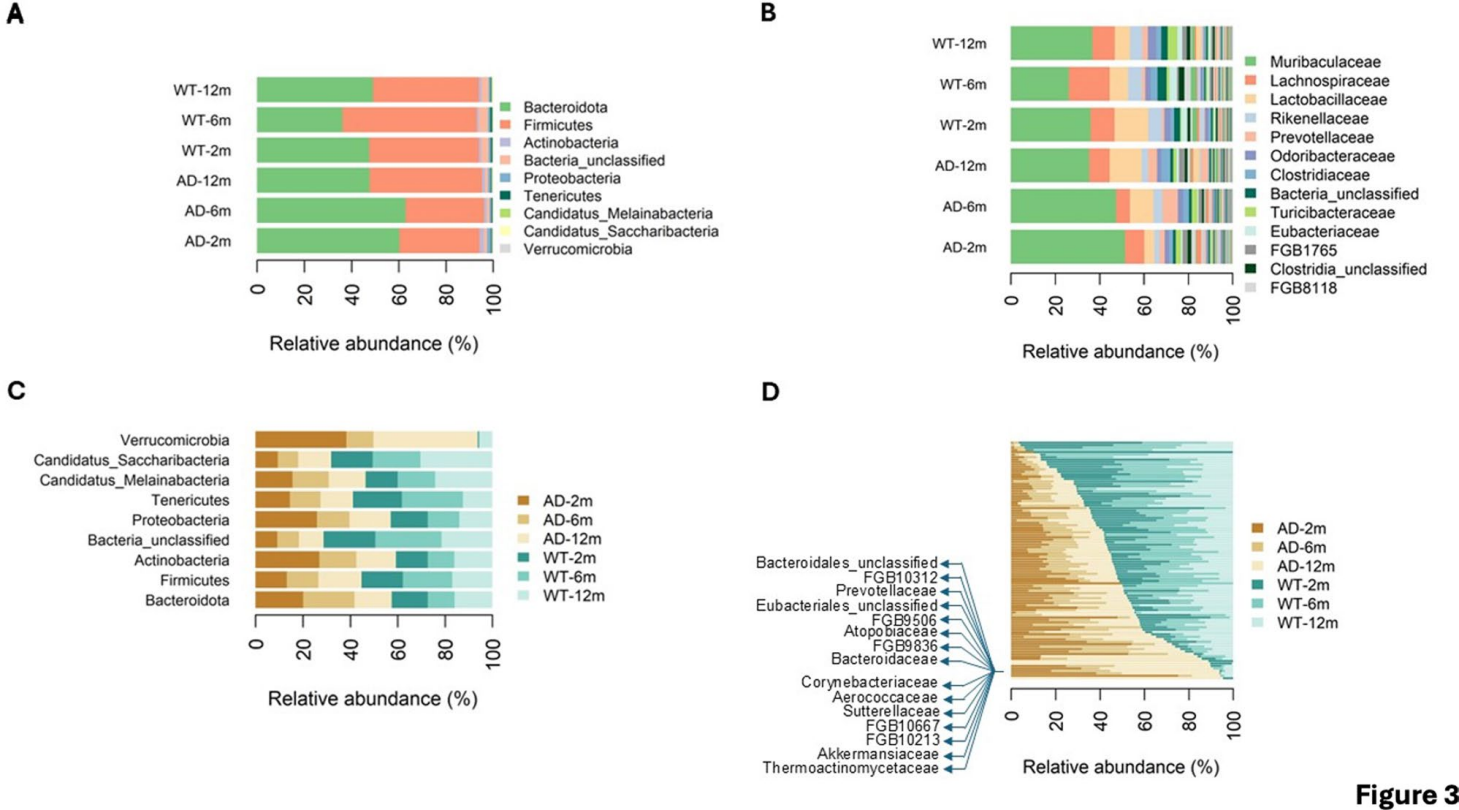
Compositional analysis of gut microbiome variations in AD and control samples at the phylum and family levels. (**A**) Bar plot showing phylum-level average relative abundances (in percent values) in AD microbiomes vs. their time-matched control counterparts. The Bacteroidota and Firmicutes phyla are predominantly observed across all conditions. On average for each condition, unclassified bacteria account for 2.3% of the relative abundances. (**B**) Bar plot showing family-level average relative abundances in AD microbiomes vs. their time-matched control counterparts. The *Muribaculaceae*, *Lactobacillaceae*, and *Lachnospiraceae* families collectively represent over 60% of the relative abundance in each condition. The color coding in the legend highlights the top 13 families ordered by average relative abundance for clarity. (**C**) Bar plot showing average relative abundances of each detected phylum across AD microbiomes and time-matched controls. (**D**) Average relative abundance of each detected family across AD microbiomes and time-matched controls. FGBs are ordered by relative abundance. Row labels for selected FGBs are shown to help identify the FGBs mentioned in the main text

At the phylum level, the majority of the microbial population in both WT and AD samples, across all time points, primarily consisted of Bacteroidota and Firmicutes (Fig. 3A). Bacteroidota accounted for an average of 51% (50.6 ± 9.75%) and Firmicutes for 44% (43.9 ± 8.97%) of the relative abundances for each condition. Additional contributions were minor, with Actinobacteria at 1.2%, Proteobacteria at 1.1%, Tenericutes at 0.48%, Candidatus Melainabacteria at 0.27%, Candidatus Saccharibacteria at 0.16%, and Verrucomicrobia at 0.11%. Furthermore, an average of 2.3% of the relative abundances was ascribed to unclassified bacteria. This microbial composition is consistent with the notion that the gut microbiota is mainly composed of four main phyla: Firmicutes, Bacteroidota, Actinobacteria, and Proteobacteria, with Firmicutes and Bacteroidota constituting the majority of the gut microbiota [16]. The most prevalent bacterial families across all samples were *Muribaculaceae* (38.8%) belonging to the Bacteroidales order, Bacteroidia class, and Bacteroidota phylum, *Lactobacillaceae* (10.6%) in the Bacilli class, and *Lachnospiraceae* (10.0%) in the Clostridia class, both belonging to the Bacillota phylum (Fig. 3B).

In comparing phylum relative abundances across samples, Verrucomicrobia, which specializes in degrading mucin—a glycoprotein found in mucus—was found predominantly in AD samples (Fig. 3C). This finding aligns with recent metagenomic analyses that have increased interest in Verrucomicrobia due to its association with various eukaryotic hosts [51] and its role in modulating immune responses and cell death [52–54].

At the family phylogenetic level, we identified 15 families that were preferentially present in AD vs. control samples, with a ratio of relative abundances between AD samples and all samples > 0.70. For instance, the *Thermoactinomycetaceae* family was almost exclusively present in AD samples—ratio of relative abundances between AD samples and all samples of 0.95—with both characterized (e.g., *Akkermansiaceae*, *Sutterellaceae*, *Aerococcaceae*, *Corynebacteriaceae*, *Prevotellaceae*) and uncharacterized (e.g., FGB10213, FGB10667, and FGB9836) families showing a tendency to be more abundant in AD vs. WT samples (Fig. 3D).

### Temporal profiling of microbiome composition in AD mice over time

**Fig. 4:**
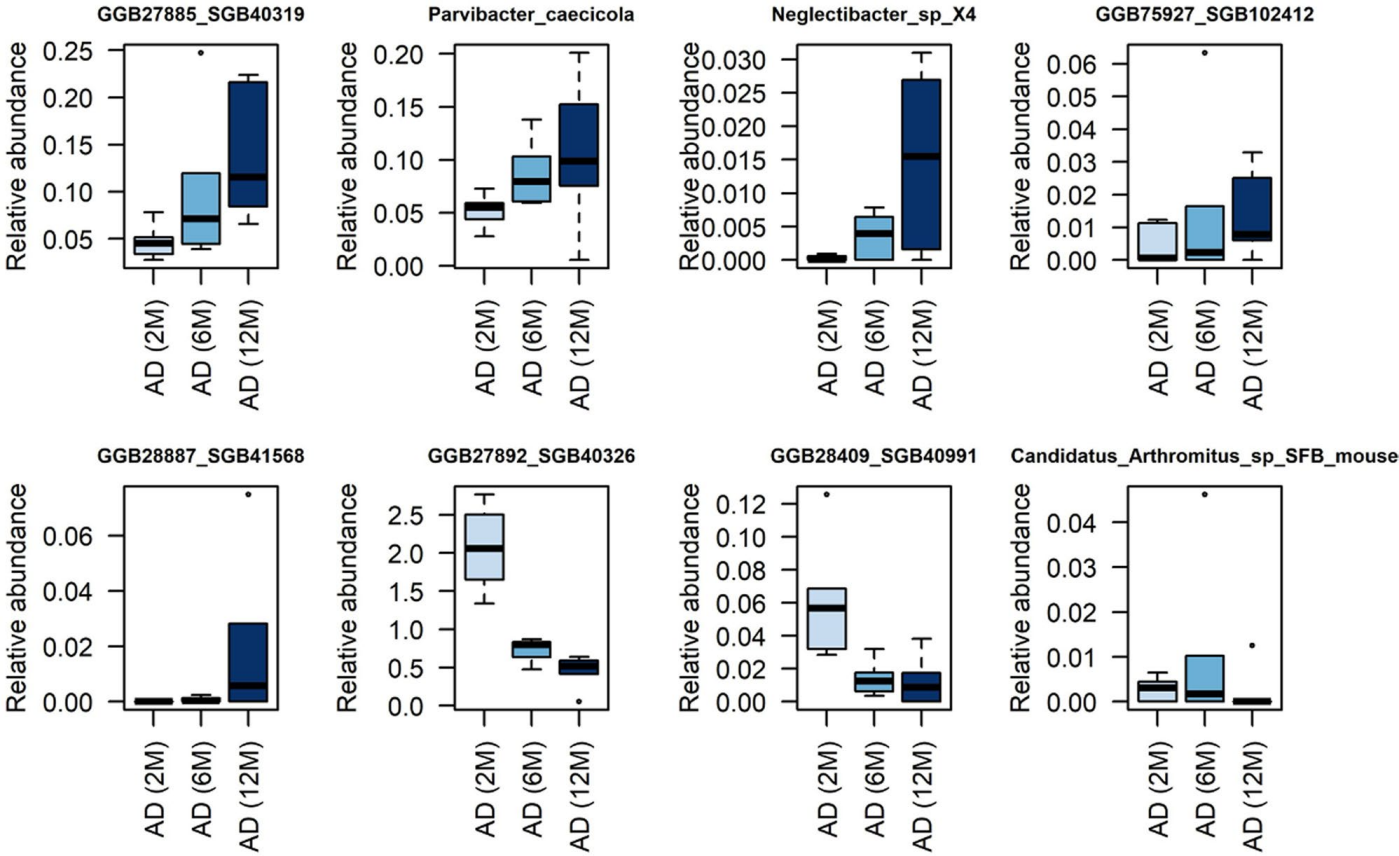
Temporal profiling of relative abundances reveals consistent trends in AD SGBs. MetaPhlAn 4 profiling unveils consistent temporal trends in a few AD microbiome species, which comprises both SGBs and those yet to be classified. Species consistently increasing with AD mouse aging include *Parvibacter caecicola* and *Neglectibacter* sp. X4, while *Candidatus Arthromitus* sp. SFB-mouse and several uSGBs display a decreasing trend over time

A preliminary analysis of the temporal profiling of microbiome compositions in AD mice revealed several species with changing abundances over time. Specifically, species such as *Parvibacter caecicola* from the *Coriobacteriaceae* family [55] and *Neglectibacter sp.* X4 of the *Oscillospiraceae* family, along with several uSGBs, showed an increase in relative abundance as the AD mice aged (Fig. 4). Conversely, a few species, including two uSGBs (i.e., SGB40326 and SGB40991) and *Candidatus Arthromitus* sp. SFB-mouse [56]—known for inducing the postnatal maturation of homeostatic innate and adaptive immune responses in the mouse gut—steadily decreased during the aging of AD mice.

Notably, species in WT samples did not display consistent patterns of increase or decrease throughout the observation periods.

### Differential abundance of genome bins between AD and control groups are dominated by taxonomically unlabeled genome bins

An initial screening to identify differentially abundant taxa between AD and control samples, as well as differential taxa across AD time points, was conducted by calculating alpha diversity statistics using Shannon’s and Simpson’s indexes. The results for each sample at each taxonomic level are summarized in Table S3. Subsequently, we performed a Wilcoxon Rank-Sum test to compare these indexes between sample groups and calculated the Bray-Curtis dissimilarity between groups as detailed in the Methods section (Table S4). Despite no evidence of distinct global modulation of microbiota perturbations between AD and WT samples or throughout the AD timeline, we decided to investigate the differential abundance of individual taxa across different ranks. Table S5 details the log2 fold change (log2FC) of each statistically significant taxon between AD and control groups.

Differential abundance analysis of SGBs between AD and WT groups at each sampled time point showed significant changes in the microbiome, with 98 SGBs showing altered levels: 56 at 2 mos of age, 38 at 6 mos, and 23 at 12 mos. Noteworthy, 75% of these differentially abundant SGBs were uSGBs, underscoring their significant contribution to the microbial gut composition. Particularly, SGB44472, SGB92816, and SGB41414 were consistently found differentially abundant at all time points. Intriguingly, a similar trend was observed for a single kSGB, *Clostridiales bacterium*—FC (T1 = 2 mos) = 20.4; FC (T2 = 6 mos) = 20.5; FC (T3 = 12 mos) = 30.0—whose ability to metabolize key amino acids precursors to neuroactive metabolites has been recently reported [57].

**Fig. 5:**
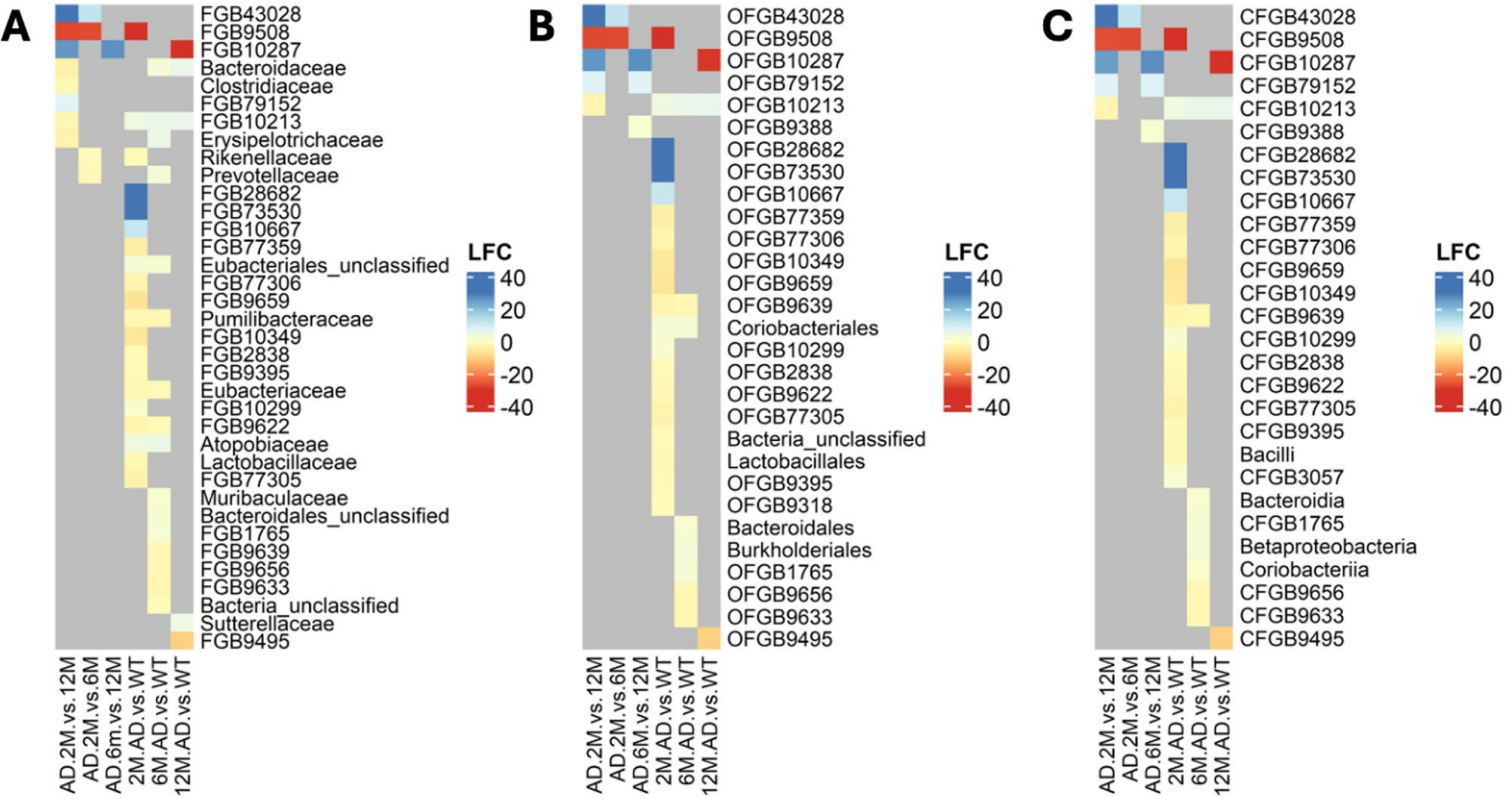
AD-associated differentially abundant genome bins at family, order, and class taxonomic ranks. The figure depicts the differentially abundant genome bins identified from two assessments: differential abundance in AD relative to control samples at each sampled time point, and differential abundance between pairs of time points (2, 6, and 12 mos) in AD samples. The heatmaps arranged from left to right display the outcomes of these differential abundance tests at the family, order, and class level, respectively. In each heatmap, column labels outline the conditions compared. The tests for differential abundance in AD samples between pairs of time points are labeled as AD.2M.vs.12M, AD.2M.vs.6M, and AD.6M.vs.12M. Time-wise differential abundance tests are labeled as 2M.AD.vs.WT, 6M.AD.vs.WT, and 12M.AD.vs.WT. Row labels indicate the taxa that were found to be differentially abundant in at least one test. Changes in relative abundance are expressed as log_2_FC. Color coding represents the intensity in fold change, with grey indicating genome bins that did not show statistically significant variations in relative abundance under the compared conditions. A genome bin is deemed differentially abundant between two conditions if it features |log_2_FC| > 1 and a Benjamini-Hochberg’s adjusted p-value < 0.05

At higher taxonomic levels, our analysis revealed 33 differentially abundant FGBs, comprising 20 uFGBs and 13 kFGBs (Fig. 5A). Furthermore, 26 order-level genome bins (OGBs) were identified as differentially abundant, with 21 uncharacterized OGBs (uOGBs) and 5 known OGBs (kOGBs) (Fig. 5B). Similarly, at the class-level, 21 out of 25 genome bins were uncharacterized (uCGBs), confirming the predominance of uCGBs prevail over known CGBs (kCGBs) (Fig. 5C).

At T1, FGBs showing decreased abundance in AD vs. WT samples outnumbered those with increased abundance, whereas at both T2 and T3 the numbers of increased and decreased FGBs were comparable. The most pronounced change in abundance were observed at T1 and T3. The largest decrease in abundance concerned FGB9508 at T1 [FC(T1 = 2 mos) = -30.0] and FGB10287 at T3 [FC(T3 = 12 mos = -30.0], while the most substantial increases were for FGB28682 and FGB73530 at T1, with both showing an FC of 30.0. Compared to uFGBs, kFGBs showed moderate changes in abundance in either direction. More precisely, *Pumilibacteraceae*, *Eubacteriaceae*, and *Lactobacillaceae* decreased at T1, whereas unclassified *Eubacteriales* and *Sutterellaceae* increased at T1 and T3, respectively. Differentially abundant kFGBs also occurred at T2, with increases observed in *Atopobiaceae* [FC(T2 = 6 mos) = 4.97], *Erysipelotrichaceae* [FC(T2 = 6 mos) = 4.60], *Bacteroidaceae* [FC(T2 = 6 mos) = 2.67], and *Muribaculaceae* [FC(T2 = 6 mos) = 1.63] and decreases in *Pumilibacteraceae* [FC(T2 = 6 mos) = -2.23] and *Eubacteriaceae* [FC(T2 = 6 mos) = -1.73]. The FGB10213 was uniquely differentially abundant in AD vs. WT samples at all time points, with FCs increasing from 3.86 to 5.15.

Differentially abundant OGBs that were taxonomically labeled included Coriobacteriales, which increased at T1 (FC = 2.51) and T2 (FC = 2.38), Lactobacillales, which instead decreased at T1 (FC = -2.02). Bacteroidales and Burkholderiales both increased at T2 (FC = 1.83, FC = 2.64, respectively). Notably, the sole OGB that consistently displayed differential abundance in AD vs. WT samples at all time points was the unclassified OFGB10213, with FCs increasing from 4.07 to 5.41. Similar to the pattern observed at the family level, the majority of statistically significant changes occurred at T1, with the most significant changes—irrespective of direction—occurring at T1 and T3. Corroborating the significance of uOGBs, the most pronounced changes were observed in OGBs lacking taxonomic labels. Specifically, the largest decrease was seen in OFGB9508 at T1 (FC = -30.0) and OFGB10287 at T3 (FC = -29.6), while the most significant increases occurred in OFGB28682 and OFGB73530, both at T1, each with an FC of 30.

Differential abundance analysis at the class level confirmed patterns recorded at lower taxonomic ranks, particularly concerning the timing of changes and the prevalence of considerable changes in uCGBs vs. kCGBs. Differentially abundant kCGBs consisted of Bacilli, which decreased at T1 (FC = -2.01). In contrast, Bacteroidia, Betaproteobacteria, and Coriobacteria all increased at T2, with FCs ranging from 1.26 to 2.62. At the phylum level, Bacteroidota increased in AD vs. WT samples at T2.

### Differentially abundant genome bins across consecutive time points

**Fig. 6:**
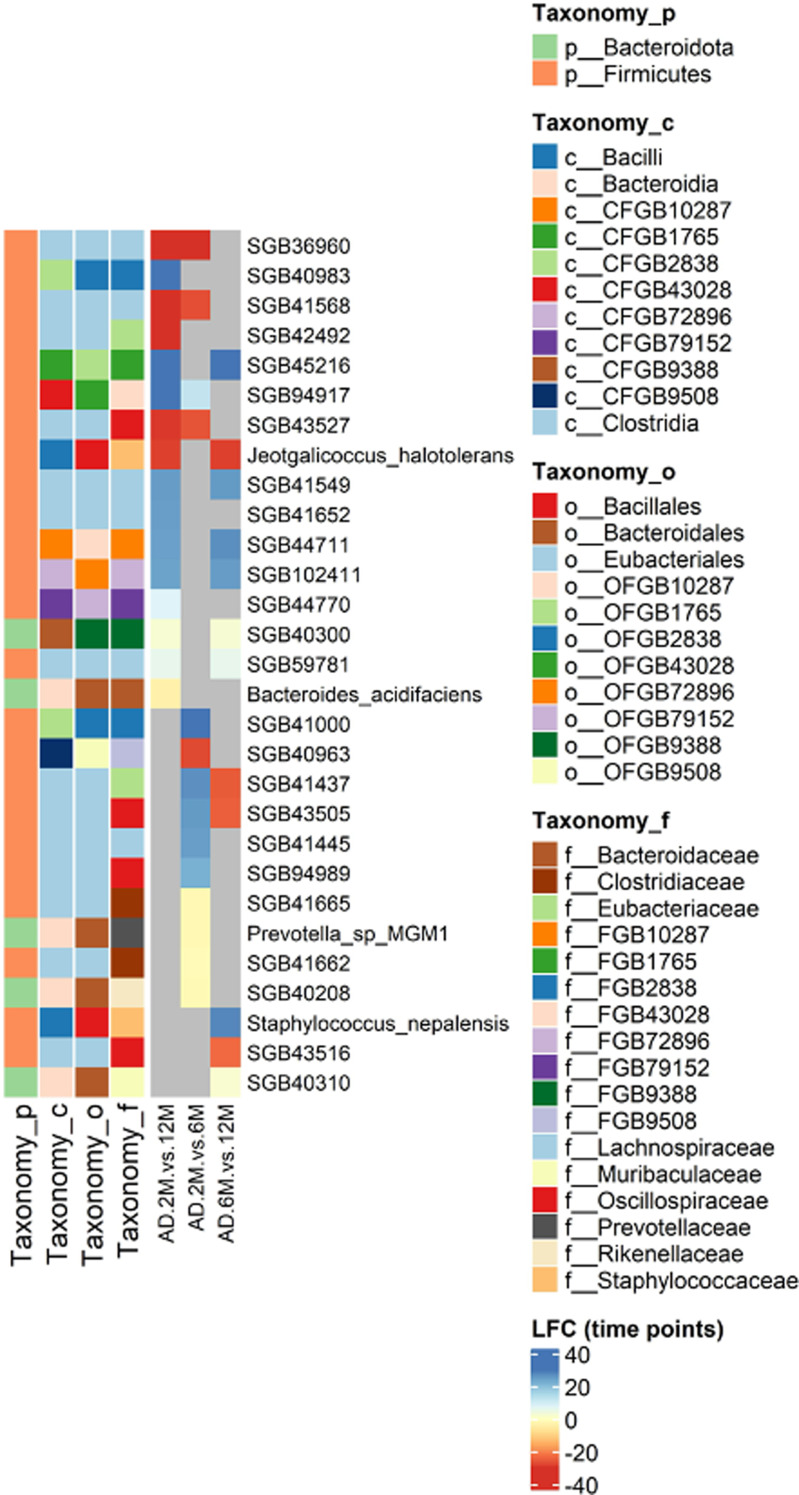
Time-associated differentially abundant SGBs. The figure shows the species genome bins that were identified as differentially abundant when comparing microbiome profiles between pairs of sampled time points (2, 6, and 12 mos) in AD samples. Differential abundance tests carried out in AD samples at T1 = 2M relative to T2 = 6M, at T1 = 2M relative to T3 = 12M, and at T2 = 6M relative to T3 = 12M are referred to as AD.2M.vs.12M, AD.2M.vs.6M, and AD.6M.vs.12M in the heatmap column labels. Row labels report the SGBs deemed differentially abundant over time. The vast majority of these SGBs do not align with any reference genome. The annotations on the left side of the heatmap categorize the differentially abundant SGBs by phylum, class, order, and family. Changes in relative abundance are reported as log2FC. Color coding indicates the intensity in fold change. Cells colored grey in the heatmap represent genome bins that did not show statistically significant changes in relative abundance between the tested temporal points. SGBs are deemed differentially abundant between two conditions if they feature a |log_2_FC| > 1 and a Benjamini-Hochberg’s adjusted p-value < 0.05

We next sought to determine how the microbial composition varied over time by computing differentially abundant taxa across each pairwise comparison among the three designated time points: T1 vs. T2, T2 vs. T3, and T1 vs. T3. Among these, a total of 29 SGBs were identified as differentially abundant, which is approximately 30% of the number observed when comparing AD to WT samples (Fig. 6). The vast majority (86.2%) of differentially abundant SGBs were not taxonomically labeled. The characterized SGBs comprised *Jeotgalicoccus halotolerans* belonging to the *Staphylococcaceae* family, Bacillales order, and Bacilli class, which increased from T2 to T3 (FC = -28.7), *Bacteroides acidifaciens* (family: *Bacteroidaceae*; order: Bacteroidales; class: Bacteroidia), which increased at T3 relative to T1 (FC = -3.48), *Prevotella sp MGM1* (family: *Prevotellaceae*; order: Bacteroidales; class: Bacteroidia), which increased from T1 to T2 (FC = -1.88), and *Staphylococcus nepalensis* (family: *Staphylococcaceae*; order: Bacillales; class: Bacilli), which decreased from T2 to T3 (FC = 28.0).

At taxonomic levels higher than species, the differentially abundant OGBs, CGBs, and FGBs were 6, 6, and 10, respectively. Out of the differentially abundant FGBs in at least a pairwise temporal comparison, kFGBs and uFGBs each accounted for 50% of the differentials. As expected, the time points showing the highest divergence in microbial composition were T1 and T3, between which the majority of statistically significant changes occurred. It should be noted that only the uFGB referred to as FGB9508 was consistently more abundant at both T2 and T3 compared to T1 (FC = -27.6 and FC = -28.0, respectively), whereas the opposite trend was observed for FGB43028 (FC = 12.1 and FC = 30.0, respectively). All kFGBs identified as differentially abundant increased either from T1 to T2, as seen in *Rikenellaceae* (FC = -1.18) and *Prevotellaceae* (FC = -1.87), or from T1 to T3, as in the case of *Bacteroidaceae*, *Clostridiaceae*, and *Erysipelotrichaceae*, with FCs ranging from − 1.93 to -3.44.

**Fig. 7:**
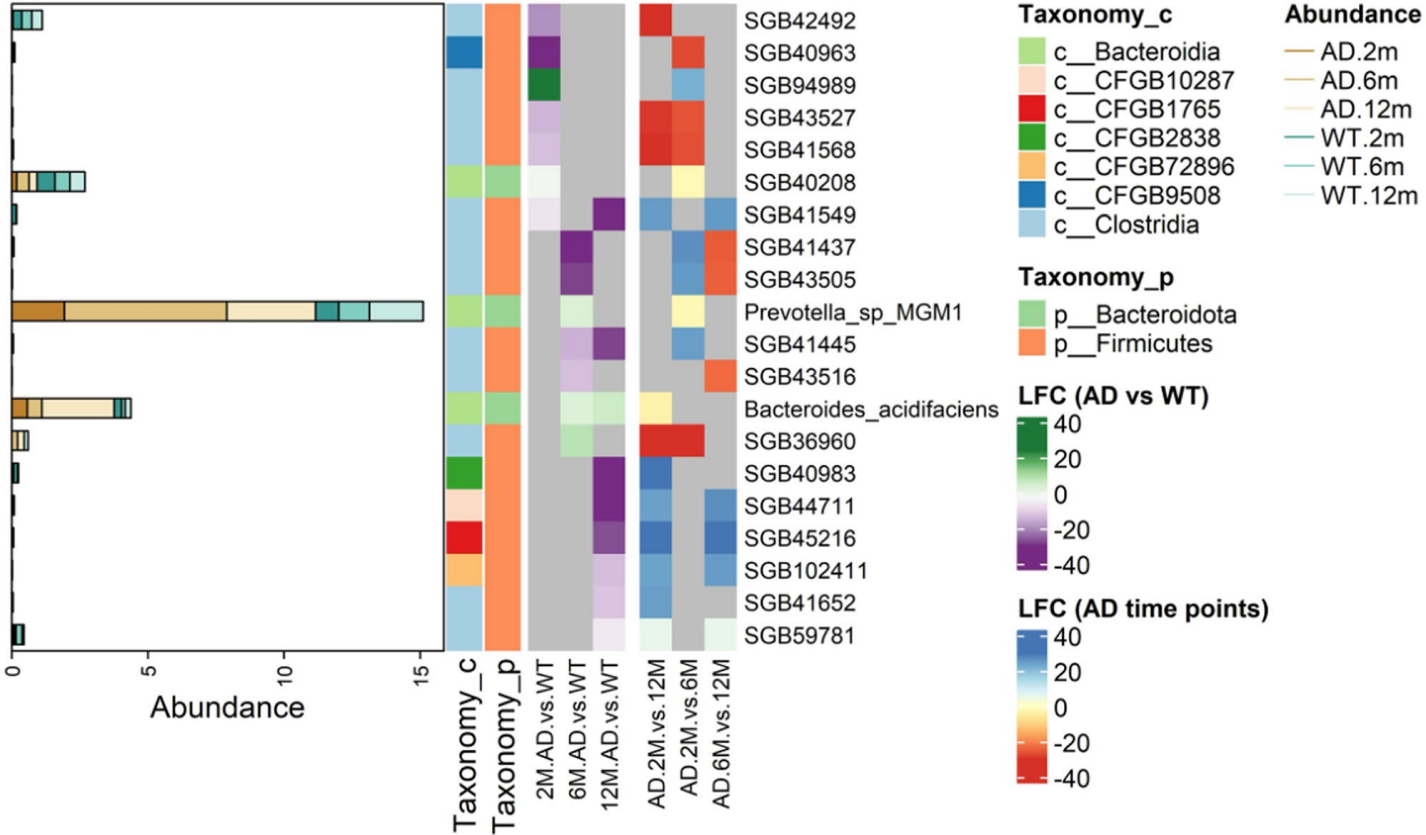
Most of the SGBs varying both between AD and WT and along AD temporal evolution are taxonomically uncharacterized. The heatmap shows the log2FC for SGBs that were differentially abundant in comparisons between AD and WT microbiomes at each sampled time point (2M.AD.vs.WT, 6M.AD.vs.WT, 12M.AD.vs.WT), and in comparisons of AD microbiomes between time points (AD.2M.vs.12M, AD.2M.vs.6M, and AD.6M.vs.12M). The differentially abundant SGBs are assigned to specific phylum and class ranks, shown in the left-sided annotation columns along with rank-specific legends. Changes in relative abundance are expressed as log_2_FC. Color coding reflects the intensity in fold change. Grey cells in the heatmap represent genome bins that did not show statistically significant changes in relative abundance between the assessed time points. An SGB is considered differentially abundant between two conditions if its |log_2_FC| > 1, and it has a Benjamini-Hochberg’s adjusted p-value < 0.05. The left-most panel shows the average relative abundance of the SGBs under each condition

We next asked which SGBs were differentially abundant both in comparisons between AD and WT samples and across the three time points in the temporal analysis of microbial composition (Fig. 7). A total of 20 SGBs appeared in both sets of analyses, with just two SGBs corresponding to taxonomically well-defined species. Specifically, *Prevotella sp. MGM1* increased its abundance in AD vs. WT samples at T2 (FC = 3.53), following an uptick from T1 to T2 in AD samples (FC = -1.88). Similarly, *Bacteroides acidifaciens* showed increased abundance in AD relative to WT samples at both T2 and T3 (FC = 3.00, FC = 5.68, respectively).

### Carbohydrate-active enzymes are widespread among marker genes of differentially abundant microbial species

**Fig. 8:**
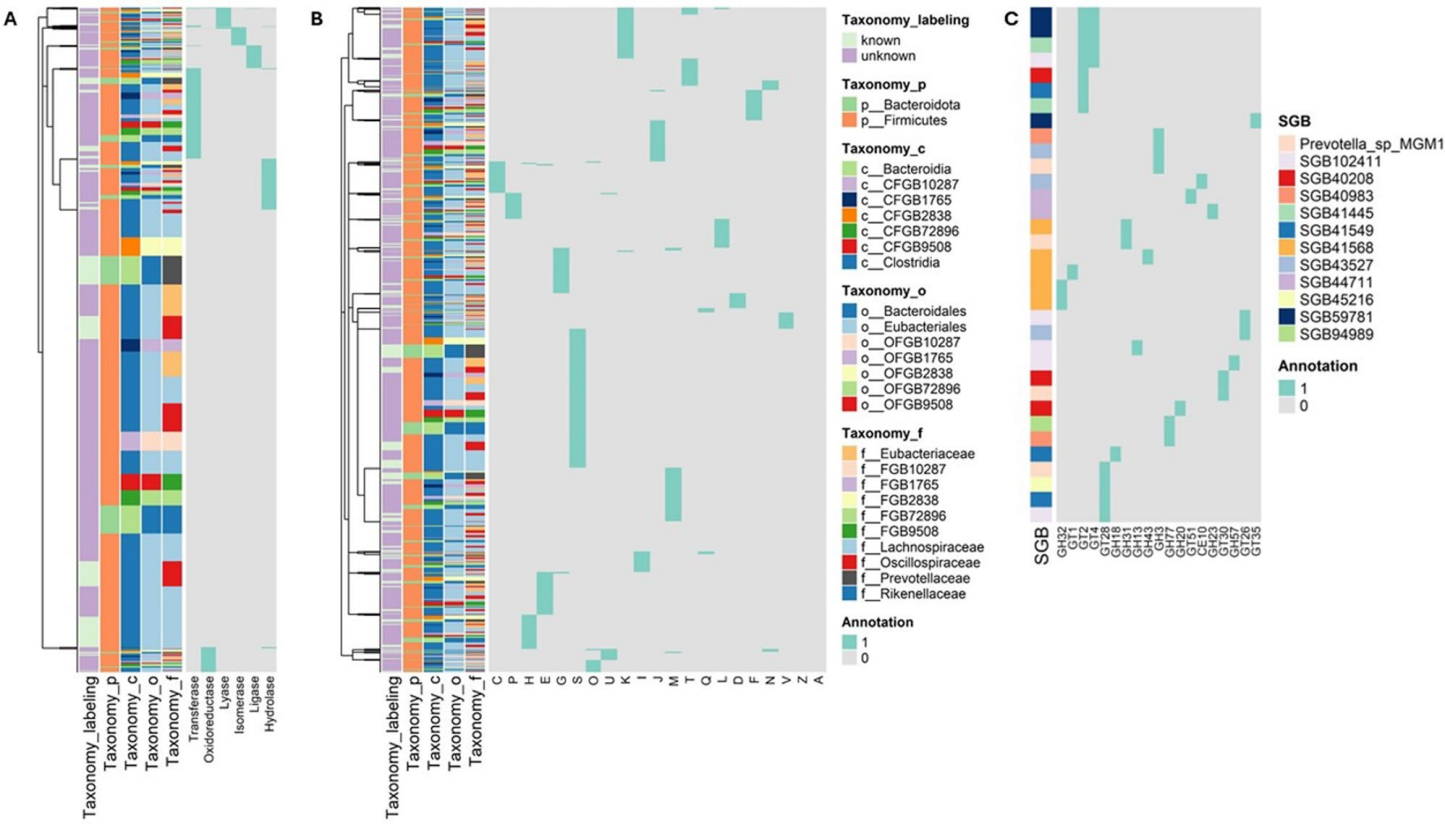
Functional profiling of unique marker genes in differentially abundant SGBs suggests untapped functional diversity in AD microbiome profiling. The figure shows the functional characterization of SGBs identified as differentially abundant in comparisons of microbial composition between AD and WT samples, as well as across AD progression stages. To this end, the marker genes associated with the selected SGBs were assembled and analyzed using various sources of functional annotation. The classification of these differentially abundant SGBs into phylum, class, order, and family ranks is shown in the left-sided annotation columns, alongside rank-specific legends. (**A**) The heatmap categorizes the unique marker genes of these SGBs based on annotations retrieved from the ENZYME database. ENZYME main classes are reported as column labels. Cells are colored grey if a marker gene lacks annotations for a specific class. (**B**) Marker genes are also categorized according to the Clusters of Orthologous Genes (COG) database. COGs are reported as column labels. Cells are colored grey if a marker gene does not have a functional assignment to a specific COG. (**C**) Categorization of marker genes uniquely characterizing the differentially abundant SGBs according to the CAZy database, which provides biochemical information on carbohydrate-active enzymes (CAZymes). CAZyme families are reported as column labels. Cells are colored grey if a marker gene is not assigned to a specific CAZyme family. The heatmaps report only those EC numbers, COG categories, and CAZyme families predicted to be associated with the marker genes

Our taxonomic profiling approach relies on SGB-specific marker genes to identify SGBs in metagenomes by mapping a sufficient fraction of these markers and by quantifying their relative abundance using within-sample-normalized average coverage estimates. Since the marker genes in the MetaPhLan database were selected by virtue of their species-specificity, we decided to use these marker gene sets per species to preliminarily detect traits of functional diversity. This analysis was particularly relevant in light of the high prevalence of uSGBs identified in our study. In particular, we focused on the SGBs classified as differentially abundant in comparisons of microbial composition between AD and WT samples and across AD progression stages. For functional annotation, we employed EggNOG-mapper, a tool designed for functional annotation based on the EggNOG database, which contains precomputed orthologs groups (OGs) covering thousands of bacterial, archaeal, and eukaryotic organisms [50]. In this analysis, the EggNOG-mapper facilitated the classification of genes using two key resources: the Clusters of Orthologous Genes (COGs) [58] and the ENZYME [59] databases. Our findings indicate that the species specificity of the marker genes, as established through coding sequence clustering, did not translate into clear functional distinctions. Remarkably, many of the marker genes from the differentially abundant SGBs were not annotated to any enzyme commission (EC) class as shown in Fig. 8A. Furthermore, a significant proportion of these genes, identified through the COG classification, was associated with unknown functions, including both kSGBs and uSGBs (Fig. 8B).

Further analysis based on the Carbohydrate-Active enZYmes database (CAZy) [60] revealed that carbohydrate-active enzymes (CAZymes) were quite prevalent among differentially abundant SGBs, accounting for 12 out of the 19 SGBs analyzed (63.1%), and representing approximately 1% of their marker genes (Fig. 8C). More precisely, the identified CAZymes mostly belonged to the families of glycoside hydrolases (GHs), which hydrolyze the glycosidic bond between two or more carbohydrates or between a carbohydrate and a non-carbohydrate moiety, and glycosyltransferases (GTs), which catalyze the transfer of sugar moieties from activated donor molecules to specific acceptor molecules, forming glycosidic bonds. The detection of these CAZymes as unique marker genes, predominantly among uSGBs, underscores the biological validity of identifying these uSGBs, highlighting the critical role of carbohydrate degradation and uptake in gut microorganisms [61].

## Discussion

Despite the recognized role of the gut microbiota in AD development and progression [4, 62] and the prevalent use of AD mouse models to investigate AD-associated gut microbiome alterations [22–25], comprehensive surveys of the gut microbiome using shotgun metagenomics across multiple time points, including comparisons with WT littermates, have been lacking.

In this study, we implemented shotgun metagenomics and developed a bioinformatic pipeline that, by employing established tools for metagenomic phylogenetic analysis of whole-metagenome shotgun samples, enabled us to conduct a comprehensive metagenomic taxonomic profiling of the gut microbiome in AD mouse models.

Our findings underscore the importance of including genomes from uncultured microorganisms in the taxonomic profiling process of AD microbiome data. Indeed, microbial species that were not identifiable through mapping against known isolate genomes constituted the largest proportion of AD- and aging-associated microbiome changes in 3xTgAD mice.

Our analysis corroborated the phylum-level composition portrayed by previous observational studies, which showed that the murine gut microbiome is predominantly composed of Firmicutes and Bacteroidota [40], which is reflected in the significant contribution of the family *Muribaculaceae* belonging to the Bacteroidota phylum.

At the family-level, our compositional analysis revealed a preferential presence of *Thermoactinomycetaceae*, *Akkermansiaceae*, and *Sutterellaceae* among kFGBs, and FGB10213, and FGB10667 among uFGBs, particularly in AD vs. WT mice.

AD samples featured a unique microbial composition distinct from age-matched WT samples. We observed that the entire phylum of Bacteroidota, as well as classes (Bacteroidia), orders (Bacteroidales), families (*Bacteroidaceae*,* Muribaculaceae*), and genera (*Muribaculum*, *Prevotella*, *Odoribacter*, *Bacteroides*, *Duncaniella*, and *Paramuribaculum*) within Bacteroidota, were more abundant in AD vs. WT samples. Bacteroidota, a diverse and abundant group of gram-negative gut bacteria [63, 64], are known for their ability to degrade complex polymers, thereby facilitating food digestion and nutrient acquisition [65]. Intriguingly, the primary component of their outer membrane, lipopolysaccharide (LPS), is known for its potential to trigger systemic inflammation, which may contribute to AD pathogenesis [66, 67]. Thus, it is tempting to speculate that the increased presence of Bacteroidota in AD samples might be linked to the development or progression of AD, underscoring their significant role in disease dynamics.

A recent meta-analysis of all genetic risk factors for AD has pointed lipid processing as a statistically enriched category of genetic risk factors for AD [68]. In particular, aged mouse microglia were shown to accumulate lipid droplets and to feature a dysfunctional state termed LD-accumulating microglia (LDAM) [69], which was also observed in a chimeric AD model [70]. Furthermore, several studies have identified certain Bacteroidetes species as major producers of short-chain fatty acids (SCFAs), which can act on brain functions via multiple mechanisms and are involved in various brain disorders, including mood disorders or autism spectrum disease [71–76]. Of note, evidence suggests that SCFAs are closely linked to AD onset and progression [77]. While these studies are mainly correlative and do not establish a clear cause-effect relationship [78, 79], SCFAs have been shown to induce biochemical changes that promote the deposition of Abeta plaques, a hallmark of AD pathogenesis [80]. It is also noteworthy that the classes of Bacilli and Coriobacteria, which respectively show decreased and increased abundance in AD vs. WT samples, are modulated by exogenous SCFAs in APP/PS1 mice [81].

As expected, the number of SGBs showing significant abundance changes between AD and healthy states was greater than that observed with aging in AD mice (98 SGBs and 29 SGBs, respectively). Notably, none of the SGB was found to be statistically significantly increased or decreased in its abundance between the early (T1) and middle (T2) AD stages, or between the middle (T2) and late (T3) AD stages. Each time point showed distinct changes in microbiome composition, predominantly involving uncharacterized SGBs and, to a lesser extent, kSGBs such as *Jeotgalicoccus halotolerans*, *Staphylococcus nepalensis*,* Bacteroides acidifaciens*, and *Prevotella sp* MGM1. Intriguingly, the latter two were found differentially abundant both in comparisons between AD and WT groups and across different time points. In particular, the Prevotella and Bacteroides genera have been identified as dominant in distinct community types in a compositional analysis of the human gut microbiome across the AD continuum [82].

The functional analysis of marker genes from uSGBs indicated that their unique gene repertoires have yet to be fully functionally categorized. Nonetheless, the detection of carbohydrate-active enzymes among these marker genes corroborates the biological relevance of these SGBs. While taxonomically labeled SGBs remain a focus of research, our findings highlight that uSGBs also warrant further investigation. Future studies investigating the links between gut microbiome and AD should include these uncharacterized microbial entities to improve our understanding of their potential roles in AD pathology. While our marker-based metagenomic analysis substantially advances our grasp of AD development and progression, a critical question remains: does the variability observed in the gut microbiome—whether comparing AD to control samples or across different AD stages—hold biological significance? To address this question, it is necessary to link specific microbiome functions to the corresponding microbial entities. Our comprehensive taxonomic profiling provides a valuable resource to further investigate the structure of the gut microbiome and its potential contributions to AD pathology and progression.

## Conclusions

In this study, we investigated the gut microbiota composition in a murine model of AD using shotgun metagenomics followed by a dedicated bioinformatic analysis grounded on a clade-specific approach, providing fresh and detailed insights into how variations in the gut microbiome composition correlate with AD development. Our research expands taxonomic characterization of the gut microbiome, revealing that a substantial part of its genomic diversity cannot be linked to known reference genomes. Our findings indicate that species lacking cultured counterparts predominantly drive the changes observed in the gut microbiome associated with AD onset and progression. Therefore, the extensive dataset generated offers a valuable resource for advancing our understanding of the complex microbial functional diversity linked to AD. This in-depth analysis not only deepens our comprehension of the microbial factors influencing AD but also sets the stage for future research aimed at uncovering potential therapeutic targets within the microbiome for treating this neurodegenerative condition.

## Electronic supplementary material

Below is the link to the electronic supplementary material.

Supplementary Material 1: Table 1- Table reporting the total number of reads obtained for each sample and the relative non-host reads.

Supplementary Material 2: Table 2- Table detailing the abundance of each taxon for each sample as measured by MetaPhlAn 4.

Supplementary Material 3: Table 3- Table providing the Shannon and Simpson indexes calculated for each sample across each taxonomic level.

Supplementary Material 4: Table 4- Table showing the significant results from the Beta diversity Adonis test between sample groups; Wilcoxon Rank-Sum Test assessing whether there is statistically significant diversity in terms of the Shannon index across the different groups, and another Wilcoxon Rank-Sum Test that assesses whether there is statistically significant diversity in terms of the Simpson index across the different groups.

Supplementary Material 5: Table 5- Table listing the log2FC of each statistically significant taxon between sample groups, as calculated by DESeq2.

## Data Availability

Raw data relative to the shotgun metagenomic experiments are available at the NCBI SRA archive, under accession id PRJNA1137132 (https://www.ncbi.nlm.nih.gov/sra/PRJNA1137132).
